# Deciding for Future Selves Reduces Loss Aversion

**DOI:** 10.3389/fpsyg.2017.01644

**Published:** 2017-09-20

**Authors:** Qiqi Cheng, Guibing He

**Affiliations:** Department of Psychology and Behavioral Sciences, Zhejiang University Hangzhou, China

**Keywords:** loss aversion multi-system theory, single-system theory, future selves, risk attitude

## Abstract

In this paper, we present an incentivized experiment to investigate the degree of loss aversion when people make decisions for their current selves and future selves under risk. We find that when participants make decisions for their future selves, they are less loss averse compared to when they make decisions for their current selves. This finding is consistent with the interpretation of loss aversion as a bias in decision-making driven by emotions, which are reduced when making decisions for future selves. Our findings endorsed the external validity of previous studies on the impact of emotion on loss aversion in a real world decision-making environment.

## Introduction

Loss aversion assumes that human beings evaluate outcomes relative to a reference point and that they tend to be more sensitive to losses than gains. This concept, initially introduced in the seminal work by Kahneman and Tversky (1979), has been one of the most widespread concepts in behavioral science and has been identified in many settings, including riskless and risky decision-making situations (Novemsky and Kahneman, 2005; Barberis, 2013). For example, in the riskless decision-making domain, loss aversion has been used to account for the endowment effect (Thaler, 1980), status quo bias (Samuelson and Zeckhauser, 1988), and in the risk domain, loss aversion has been used in the equity premium puzzle (Benartzi and Thaler, 1995) as well as to account for disposition effects (Weber and Camerer, 1998), framing effects (Tversky and Kahneman, 1981), and many others (Camerer, 2005; Barberis, 2013). Overall, loss aversion is one of the most well-established departures from the canonical expected utility model and is commonly viewed as an irrational bias (Barberis, 2013). Therefore, it is of great importance to identify its foundations.

Despite numerous empirical studies supporting the existence of loss aversion, the underlying sources of loss aversion are still not agreed upon (Ariely et al., 2005; Novemsky and Kahneman, 2005; Walasek and Stewart, 2015). For example, whether loss aversion is the by product of a single-system or a result of the interaction of multiple systems within the brain is still under debate among scholars (Rick, 2011). The multi-system theory adopts the general idea that the aversive response to loss originating from the hot system tends to interact with the more objective evaluation performed by the cool system (Ashraf et al., 2005; Camerer et al., 2005). This theory emphasizes the role of negative emotions, such as fear or anxiety, in enhanced sensitivity to losses. Alternatively, a single-system model suggests that loss aversion could reflect an asymmetric response to losses versus gains within a single-system that codes for the subjective value of the item (Knutson et al., 2003; Tom et al., 2007). This theory suggests that loss aversion is adequately explained by a single-system that treats gains and losses asymmetrically. However, both theories appear to have their supporting evidence, as reviewed in Rick (2011). Therefore, more empirical studies are needed to discriminate between those two theories (Keren and Schul, 2009).

The core of the debate is whether a negative emotion is necessary for loss aversion. However, answering this question in a precise laboratory setting is difficult because we cannot provide a decision situation in which participants can make choices without any influence of emotions. First, a background negative emotion cannot be fully eliminated. For example, magnetic resonance imaging (MRI) scans frequently trigger a state of anxiety in individuals being scanned (Chapman et al., 2010). These background emotions have an impact on decision-making, so we cannot ensure that they have no impact on loss aversion (see review by Phelps et al., 2014; Lerner et al., 2015). Second, for a new set of similar decisions with the same structures, emotions may be aroused only in the first several trials, which works together with the evaluation system to set a loss aversion parameter and stores that parameter in the valuation system suggested by Tom et al. (2007). The percent of emotionally aroused trials is so small that they can be ignored by the fitting model used to analyze the neuro data. Past emotional experience utility may also help to set the parameter, but this effect also could not be observed in the lab. In these cases, we cannot say that emotion is not necessary for loss aversion. Third, communications between multi-structures are not always detected by our scanners when their signals are weak. For example, the connectivity between the amygdala and striatum is too close and complex, so this closeness and complexity may prevent weak communications from being detected using the current technology. Therefore, a feasible way to respond to the debate now is to determine whether a different emotional intensity will lead to a significantly different degree of loss aversion for the same decisions.

In this study, we distinguished the single-system and multi-system explanations of the loss aversion by investigating whether making decisions for future selves reduces loss aversion. When people make decisions for their future selves, the outcome-related and action-related emotions associated with decision-making are less intense compared when they make decisions for their current selves (Kassam et al., 2008; Lerner et al., 2015). In this decision situation, a single-system theory predicts that the degree of loss aversion when deciding for their future selves should be no different from when they decide for their current selves. In contrast, the multi-system theory predicts that—due to reduced emotional intensity when making decisions for future selves—participants who make decisions for the future will be less loss averse compared to those who make decisions for their current selves. We compared the degree of loss aversion when participants make decisions for their future selves to when participants make decisions for their current selves. We found that deciding for future selves reduces loss aversion. Our findings favor the multi-system explanation as the most appropriate for loss aversion between the two explanations.

## Materials and Methods

### Participants

Two hundred eighty-five undergraduate participants aged 17∼23 (157 females and 128 males) were recruited from classes at Zhejiang University (China), and eight experimental sessions were conducted in the classroom. Since all participants played independently, all 285 decisions are independent observations. The Research Ethics Board of Zhejiang University approved the current study. All participants provided written informed consent before participating in the experiment.

### Experimental Design

Suppose that a participant can choose between $ 30 with certainty and a lottery (100, 40%; 0, 60%). In one case, L (L for loss, lottery L), the participant needs to pay $ 30 for the choice. In another case, G (G for gain, lottery G), the participant has the choice for free. In terms of expected value, the choices in the two cases are the same: in each choice, the probabilities, their potential outcomes, and the expected value difference between the two options are identical. A participant without loss aversion should treat the choices in these two cases as the same. More specifically, the willingness to choose the risky prospect in L is supported by many factors, including loss aversion, risk preference, impulsivity, and many others (Köbberling and Wakker, 2005). In the case of G, the effect of loss aversion on the willingness to choose the risky prospect will not exist anymore, and other factors influencing the willingness are the same as those in the case of L. Therefore, the difference in the willingness to choose the risky prospect between the lotteries in L and G can be attributed to loss aversion, with its magnitude reflecting the degree of loss aversion (Morrison and Oxoby, 2014). For example: willingness to choose the risky prospect (lottery L) = risk preference + impulsivity + other factors + loss aversion. Willingness to choose the risky prospect (lottery G) = risk preference + impulsivity + other factors. Loss aversion = Willingness to choose the risky prospect (lottery L) – Willingness to choose the risky prospect (lottery G).

To measure the difference between lottery L and G for both current and delayed situations in the willingness to choose the risky prospect, we employ a 2(Future (delayed lotteries) vs. Now (instant lotteries)) × 2(Gain vs. Loss) between-subjects mixed experimental design^1^. Therefore, we have four treatments: “Future Gain,” “Future Loss,” “Now Gain,” and “Now Loss” (FG, FL, NG, NL, henceforth). The difference in willingness to take the risk between FG and FL indicates the degree of loss aversion for the future, and the difference between NG and NL indicates the degree of loss aversion at the present.

In fact, when people make decisions for their future selves, the willingness to choose the risky prospect in case L for the future might have an additional contributing factor: the sign effect (the gain/loss asymmetry in the discounting rate). The sign effect encourages people to make a safe choice for the future, which is the same as the effect of loss aversion. Because the sign effect and loss aversion both have a negative effect on the willingness to choose the risky prospect, their impact on the willingness to choose the risky prospect in the loss domain will not offset each other. In fact, as discussed later in Section “Discussion,” the sign effect is suggested as the result of loss aversion in the intertemporal choice tasks. Therefore, the impact of the sign effect is the impact of loss aversion, which will not affect our measurement of the impact of loss aversion for the future treatment.

### Tasks and Procedure

To measure the willingness to choose the risky prospect, we follow Holt and Laury (2002, 2005) decision task by asking participants to make a series of binary choices for 20 pairs of options (**Table 1**). The first option (Option A, the safe option) in each pair is always RMB 10 (10 Chinese Yuan) with certainty. The second option (Option B, the risky option) holds the potential outcomes constant at RMB 18 or 1 for each pair but changes the probabilities of winning for each decision, which creates a scale of increasing expected values. Because expected values in early decisions favor Option A while the expected values in later decisions favor Option B, an individual should initially choose Option A and then switch to Option B. Therefore, there will be a ‘switch point,’ which reflects a participant’s willingness to choose a risky prospect. The participants are told that each of their 20 decisions in the table has the same chance of being selected and their payment for the experiment will be determined by their decisions.

**Table 1:** The 20 paired incentivized lottery-choice decisions.

Decision	Option A	Option B
1	100% of 10 Yuan	1/20 chance of 18 Yuan and 19/20 chance of 1 Yuan
2	100% of 10 Yuan	2/20 chance of 18 Yuan and 18/20 chance of 1 Yuan
3	100% of 10 Yuan	3/20 chance of 18 Yuan and 17/20 chance of 1 Yuan
4	100% of 10 Yuan	4/20 chance of 18 Yuan and 16/20 chance of 1 Yuan
5	100% of 10 Yuan	5/20 chance of 18 Yuan and 15/20 chance of 1 Yuan
6	100% of 10 Yuan	6/20 chance of 18 Yuan and 14/20 chance of 1 Yuan
7	100% of 10 Yuan	7/20 chance of 18 Yuan and 13/20 chance of 1 Yuan
8	100% of 10 Yuan	8/20 chance of 18 Yuan and 12/20 chance of 1 Yuan
9	100% of 10 Yuan	9/20 chance of 18 Yuan and 11/20 chance of 1 Yuan
10	100% of 10 Yuan	10/20 chance of 18 Yuan and 10/20 chance of 1 Yuan
11	100% of 10 Yuan	11/20 chance of 18 Yuan and 9/20 chance of 1 Yuan
12	100% of 10 Yuan	12/20 chance of 18 Yuan and 8/20 chance of 1 Yuan
13	100% of 10 Yuan	13/20 chance of 18 Yuan and 7/20 chance of 1 Yuan
14	100% of 10 Yuan	14/20 chance of 18 Yuan and 6/20 chance of 1 Yuan
15	100% of 10 Yuan	15/20 chance of 18 Yuan and 5/20 chance of 1 Yuan
16	100% of 10 Yuan	16/20 chance of 18 Yuan and 4/20 chance of 1 Yuan
17	100% of 10 Yuan	17/20 chance of 18 Yuan and 3/20 chance of 1 Yuan
18	100% of 10 Yuan	18/20 chance of 18 Yuan and 2/20 chance of 1 Yuan
19	100% of 10 Yuan	19/20 chance of 18 Yuan and 1/20 chance of 1 Yuan
20	100% of 10 Yuan	20/20 chance of 18 Yuan and 0 chance of 1 Yuan

*Below are 20 decisions, each of which has an equal chance of being used to determine your final earnings in **one month (vs. now) (*bold*).** That is, you make your decisions now, and the settlement of profit and loss will occur in **one month (vs. now).** Each decision corresponds to a lottery, which will **cost you RMB 10 Yuan (vs. will not cost you anything).** You can choose between Option A: a sure gain of RMB 10 and Option B: *p* chance to earn RMB 18 and *1-p* chance to earn RMB 1. You can choose your preferred option, and your decision will determine your profit and loss in **one month (vs. now**). Please make your choice*.

All experimental sessions are conducted in the classroom, not a laboratory^2^. Before the incentivized section, each participant completes a hypothetical version of the risk preference elicitation decision table (see **Table 2**) to control for individual variation^3^. Additionally, this hypothetical section is introduced for another two reasons. The first reason is that we must ensure that the participants understand the rules of the task; the second reason is that we must ensure that they have the ability to calculate the expected amount of each option in the incentivized section. The calculation of the expected utility of each choice is more difficult than in the incentivized section, and participants who easily complete this task will also find the incentivized task easy.

**Table 2:** The 20 paired hypothetical lottery-choice decisions.

Decision	Option A	Option B
1	1/20 chance of $10; 19/20 chance of $8	1/20 chance of $19; 19/20 chance of $0.5
2	2/20 chance of $10; 18/20 chance of $8	2/20 chance of $19; 18/20 chance of $0.5
3	3/20 chance of $10; 17/20 chance of $8	3/20 chance of $19; 17/20 chance of $0.5
4	4/20 chance of $10; 16/20 chance of $8	4/20 chance of $19; 16/20 chance of $0.5
5	5/20 chance of $10; 15/20 chance of $8	5/20 chance of $19; 15/20 chance of $0.5
6	6/20 chance of $10; 14/20 chance of $8	6/20 chance of $19; 14/20 chance of $0.5
7	7/20 chance of $10; 13/20 chance of $8	7/20 chance of $19; 13/20 chance of $0.5
8	8/20 chance of $10; 12/20 chance of $8	8/20 chance of $19; 12/20 chance of $0.5
9	9/20 chance of $10; 11/20 chance of $8	9/20 chance of $19; 11/20 chance of $0.5
10	10/20 chance of $10; 10/20 chance of $8	10/20 chance of $19; 10/20 chance of $0.5
11	11/20 chance of $10; 9/20 chance of $8	11/20 chance of $19; 9/20 chance of $0.5
12	12/20 chance of $10; 8/20 chance of $8	12/20 chance of $19; 8/20 chance of $0.5
13	13/20 chance of $10; 7/20 chance of $8	13/20 chance of $19; 7/20 chance of $0.5
14	14/20 chance of $10; 6/20 chance of $8	14/20 chance of $19; 6/20 chance of $0.5
15	15/20 chance of $10; 5/20 chance of $8	15/20 chance of $19; 5/20 chance of $0.5
16	16/20 chance of $10; 4/20 chance of $8	16/20 chance of $19; 4/20 chance of $0.5
17	17/20 chance of $10; 3/20 chance of $8	17/20 chance of $19; 3/20 chance of $0.5
18	18/20 chance of $10; 2/20 chance of $8	18/20 chance of $19; 2/20 chance of $0.5
19	19/20 chance of $10; 1/20 chance of $8	19/20 chance of $19; 1/20 chance of $0.5
20	20/20 chance of $10; 0/20 chance of $8	20/20 chance of $19; 0/20 chance of $0.5

*Imagine that you must make the following decisions. First, examine both options, then indicate the options you prefer. All payoffs from this section are hypothetical and will not be paid to you*.

### Data

In all our four treatments, in the first decision, only an extreme risk taker would choose Option B. When the probability of the high payoff outcome increases sufficiently (moving down the table), a person should change over to Option B. Five participants switched back from B to A in either the hypothetical or incentivized section, and three participants always chose Option A, even in the last decision. Those eight participants have been excluded from the analysis because their decision data cannot be used for our purposes. Other reasons also support this exclusion: the first is that this anomaly is not caused by loss aversion. After the experiment, we asked those participants about their choices, and we found that this type of switching back in our experiment came from a misunderstanding of the incentive structure. The second reason is that only 8 out of 285 were excluded, which is less than 3%. Their choices have no effect on the result of this research, whatever they chose. Therefore, we have 277 valid observations. Brief statistics are shown in **Table 3**.

**Table 3:** Brief statistics.

Treatment	Number of observations	Mean switch point of the incentivized decision section	Mean switch point of the hypothetical decision section
NG (Now, Gain)	68	12.56 (3.633929)	11.38235 (3.29644)
NL (Now, Loss)	69	14.38 (3.59371)	10.73913 (3.41128)
FG (Future, Gain)	73	12.16 (3.75283)	11.0411 (3.80584)
FL (Future, Loss)	67	11.10 (3.43835)	10.47761 (3.14478)

## Results

**FIGURE 1:**
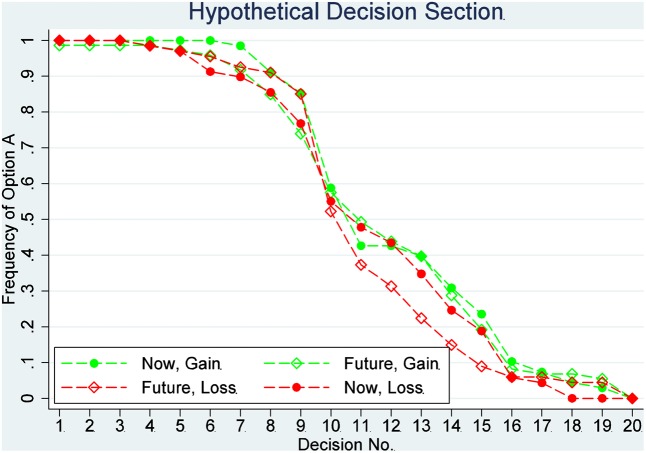
Proportion of safe choices in each decision: the hypothetical decision.

**FIGURE 2:**
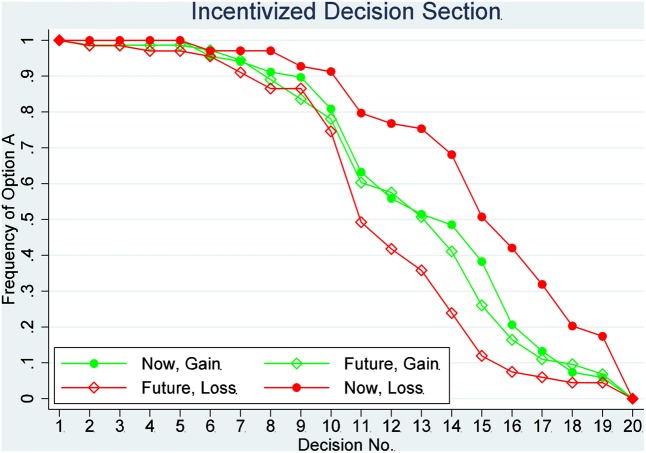
Proportion of safe choices in each decision: the incentivized decision section.

The switch point for a participant is defined as the number of Option A she/he chose if and only if there is one switch. The proportion of safe choices in each decision is shown in **Figures 1**, **2**. **Figure 1** shows the results of the incentivized section, and **Figure 2** shows the results of the hypothetical section. The effects of delay and loss on the switch point are assessed by a two-way ANOVA. Tukey multiple comparisons of means with a 95% family-wise confidence level are provided later, which can be used to determine which means amongst these four means differ from the rest. The means of the switch point are also compared using *post hoc* two-sample *t*-tests for independent variables.

The assumption that the data from these four treatments in the incentivized section are comparable is supported by the outcomes in the hypothetical section. The two-way ANOVA analysis for the hypothetical section indicated no significant main effect of delay (*F* = 6.284, *P* = 0.4657) or loss (*F* = 2.14, *P* = 0.1448) and no significant interaction between delay and loss (*F* = 0.01, *P* = 0.9231). The participants in those four groups do not have different mean switch points in the hypothetical section. Therefore, we cannot reject the hypothesis that participants in those four treatments are drawn from the same population (a Kruskal–Wallis test confirms this result, *P* = 0.7093). All participants in those four treatments show weak risk aversion in the hypothetical section, with an average switch point of 10.97. This is significantly larger than 9, which is a switch point that a risk-neutral participant would choose (one sample *t*-test, *P* = 0.0000).

**FIGURE 3:**
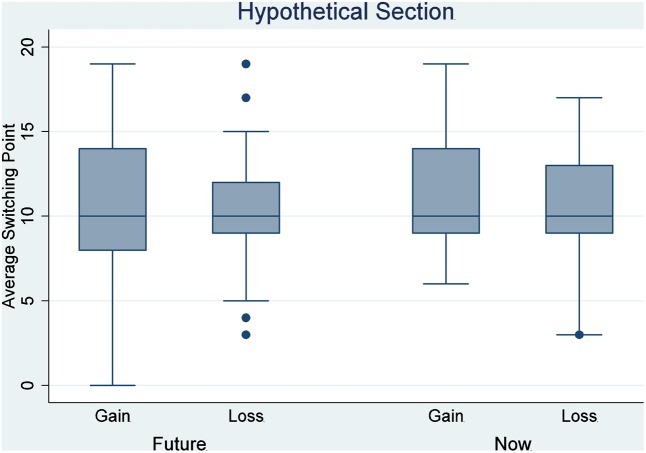
Average switch point by treatment: the hypothetical decision section.

**FIGURE 4:**
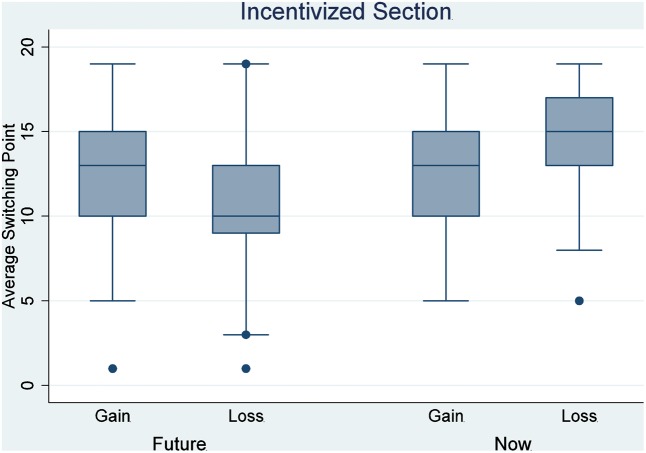
Average switch point by treatment: the incentivized decision section.

The two-way ANOVA analysis for the incentivized section indicated a significant main effect of delay on the switch point (*F* = 17.550, *P* < 0.001), and a significant interaction between delay and loss (*F* = 10.993, *P* = 0.00104). The main effect of loss is not significant (*F* = 0.706, *P* = 0.40139). A Tukey multiple comparison of means shows no significant difference in the average switch points between any two of the three treatments, NG, FG, and FL (*P* > 0.09 for all these three comparisons), but the average switch point in NL was significantly different from the average switch point in any of the other three treatments (*P* < 0.05 for all comparisons with the other three treatments). Therefore, we can reject the hypothesis that the responses of individuals in those four treatments are drawn from the same distribution. More specifically, there is a significant difference between NG treatment and NL treatment (Tukey multiple comparisons of means, *P* = 0.0181888). That is, relative to the gain condition, the participants in the loss treatment were unwilling to give up the RMB 10 in their possession to participate in a lottery, accordingly requiring significantly larger returns to bear the risk of the lottery. This behavior is thus consistent with loss aversion. On the contrary, there is no significant difference between FG and FL treatment (Tukey multiple comparisons of means, *P* = 0.3073135). Therefore, we cannot reject the null hypothesis that these responses are drawn from the same distribution. This result suggests that loss aversion may not be available when participants make decisions for the future. The average switch points in those four treatments are shown in **Figures 3**, **4**; **Figure 3** shows data from the hypothetical section, and **Figure 4** shows data from the incentivized section.

As explained in the experimental design section, the willingness to choose risky prospects in the loss condition minus that in the gain condition reflects the degree of loss aversion. The data for the willingness to choose risky prospects in all four conditions is normally distributed (skewness and kurtosis test for normality, *P* > 0.1 for all four treatments). Because any linear combination of independent normal deviates is a normal deviate, loss aversion that comes from the linear transformation from an independent normal distributed willingness to choose risky prospects will be normally distributed. The data shows that the degree of loss aversion in the “Now” condition is significantly larger than that of the “Future” condition (two-sample *t*-test, *P* = 0.0006). Therefore, we infer that making decisions for future selves reduces loss aversion.

## Discussion and Conclusion

The goal of the current study was to distinguish the single-system and the multi-system explanations of loss aversion at the behavioral level in a real world decision-making environment. We compared the degree of loss aversion when they make decisions for current and future selves. Because one’s emotional response to reward in the future treatment is less intense than that in the current treatment (Kassam et al., 2008; Lerner et al., 2015), the difference of the loss aversion degree between the current and future treatment reflects the impact of emotion on the degree of loss aversion. In this setting, the multi-system theory predicts that making decisions for future selves reduces loss aversion. In contrast, the single-system theory predicts that making decisions for future selves will not reduce loss aversion. We find that participants who make decisions for future selves — with less emotional intensity when making decisions for future selves — are less loss averse compared to those who make decisions for their current selves. This finding is consistent with the prediction given by the multi-system theory. Therefore, our results favor the hypothesis of the multi-system theory but disfavor the hypothesis of the single-system theory. Our results also confirm the debiasing effect of making decisions for the future.

Our findings are consistent with previous reports that emotion influences loss aversion. The idea that emotions are used to inform judgments and decisions has been investigated for decades (Loewenstein, 1996, 2000; Bechara et al., 2000; Loewenstein et al., 2001; Loewenstein and Lerner, 2003; Schwarz, 2012). Recently, the impact of emotions on loss aversion has been asserted, which hypothesized that loss aversion is an expression of fear (Camerer, 2005; Hartley and Phelps, 2012). Increasingly, the literature shows that emotion plays a critical role in loss aversion (Isen et al., 1988; Schulreich et al., 2014). For example, Sokol-Hessner et al. (2013) found that emotion regulation can reduce loss aversion. Schulreich et al. (2016) found that even incidental fear cues influence loss aversion. Our study adds to this literature by providing evidence of the impact of emotions on loss aversion in a more realistic decision-making environment. In our study, the participants do not need to complete their decisions in a short amount of time (a fast decision in less than 5 s), which is required in most of the previous studies. Therefore, the current study endorsed the external validity for previous studies that confirm the role of emotions role in loss aversion in this line. Our finding is also consistent with previous studies concerning decision-making on behalf of others, which found that making decisions for others reduces loss aversion (Andersson et al., 2014). Our study can provide more solid evidence for the multi-system theory than those previous studies in this line because the future losses in our study are the decision makers’ own losses. Together with studies concerning the impact of emotions on loss aversion, our current study favors a multi-system theory.

Concerning the neural mechanisms, whether the structures that possess affections, such as amygdala and insula, play a key role in loss aversion is still under debate. The single-system theory posits that these structures have no effect on loss aversion, while the multi-system theory posits that these structures play a key role in loss aversion. The single-system explanation is supported by the evidence observed by Tom et al. (2007), in which the authors found a unified representation of expected utility (which included loss aversion) in VMPFC and striatal responses (among other regions but not the amygdala). In addition, studies on related topics such as the frame effect (Li et al., 2017) and the hyperbolic discounting (Kable and Glimcher, 2007) also show that these biases can be observed without the presence of emotion activity. The multi-system explanation is supported by De Martino et al. (2010), in which participants with bilateral amygdala lesions showed a dramatic absence of loss aversion when they retained a normal response to the reward magnitude. After this study, recent fMRI studies also showed that the amygdala and the insula are involved in loss aversion (Canessa et al., 2013; Sokol-Hessner et al., 2013; Canessa et al., 2017), and both structures are thought to be central to effective processing (Phelps et al., 2001; LeDoux, 2003; Paulus and Stein, 2006; Kim et al., 2011). Kurnianingsih and Mullette-Gillman (2016) also find evidence of significant differences in the neural processing of gains and losses when studying the neural basis of loss value coding. Obviously, different conclusions come from different experimental designs, tasks, and procedures. In most fMRI studies, the participants must make 100s of decisions to have only several paid (for example, only 3 trials will be chosen for payment in 256 trials). Each trial has a small effect on the final reward. Encoding the potential loss is a cost for participants (Basten et al., 2010; Lerner et al., 2015), so they not only dislike the loss but also the anticipated disutility (negative emotions related to loss). Therefore, it is “ecologically irrational” to encode loss in the amygdala in every trial if there are plenty of decisions with the same structure (Goldstein and Gigerenzer, 2002). When the potential payoff related to each trial is small and repeated, there must be an alternative way to encode them rather than to fully encode them in the amygdala, which could reduce the perceived negative emotions. A possible mechanism is that for a new set of similar decisions with the same structures, emotions may be aroused only in the first several trials, which works together with the valuation system (VMPFC and striatum) to set the loss aversion parameter and stores that parameter in the valuation system (VMPFC and striatum), the single valuation system suggested by Tom et al. (2007). Another rational way for a participant to avoid negative emotions is to find and use a fixed decision rule. For example, in Li et al. (2017)’s study, the participants can obey a simple rule of always choosing the option that has more green parts among the two options (see Figure 1 of Li et al., 2017), which leads to a consistent choice. At the same time, habituation of the neural response to repeated stimuli has been well-demonstrated (Fischer et al., 2003). All these reasons may contribute to the undetected emotion activity in Tom et al. (2007) and Li et al. (2017) studies. Thus, a possible story for the conflicts is that in the beginning, multiple systems work together to set the degree of loss aversion and store that information in the valuation system. Thus, the single-system theory may capture this latter part of the whole story, the part that shows how the stored loss aversion parameter affects evaluation. In contrast, the multi-system theory emphasizes the first part of the story that describes the role of emotions in setting and storing the loss aversion parameter. This finding highlights the role of emotions in setting the degree of loss aversion.

Our conclusion that deciding for the future reduces loss aversion will not be altered by the impact of the sign effect on the willingness to choose the risky prospect in the decision. The sign effect, which is unanimously confirmed in intertemporal choice tasks, shows that people discount future gains more than future losses (Frederick et al., 2002). This asymmetry, if it exists in our study, will affect the willingness to choose the risky prospect in a future lottery that contains both potential loss and gain. In a future lottery that only contains gain, the willingness to choose the risky prospect will not be affected by the sign effect, even if it exists. Thus, the difference of the willingness to take risks between the two future lotteries with and without loss might be affected by the sign effect. In fact, both the sign effect and loss aversion have a positive impact on the difference. Therefore, the reduced willingness to choose the risky prospect in the future treatment compared to that in the current treatment should not be attributed to the sign effect but should instead be attributed to reduced loss aversion. Additionally, previous fMRI studies concerning the sign effect suggest that loss aversion may be one of the main causes of the sign effect. This explanation is consistent with recent studies that examined the neural difference between delay discounting of gains and losses (Bickel et al., 2009; Xu et al., 2009; Tanaka et al., 2014; Zhang et al., 2016). This explanation suggests that the sign effect may not work in our current study. The possible reason is that the remote loss in the future lottery in our task is not accompanied by any immediate loss. In the intertemporal discounting tasks, the participants have to choose between an immediate loss and a remote loss, in which the remote loss is always accompanied by an immediate loss. The negative emotion related to the immediate loss will act as an incidental emotional state for the remote loss, inducing loss aversion for the remote loss and then a sign effect. In contrast, the future loss has no connection with such emotion in our study. Therefore, it is acceptable that both sign effect and loss aversion being reduced in this study and the reduced willingness to choose the risky prospect be attributed to reduced loss aversion.

Our finding supported the multi-system theory at the behavioral level, and we proposed a possible explanation of the whole story of initiating and storing the loss aversion parameter among multi systems. However, a clear limitation of this study is that we do not provide any neurological evidence to adequately support our explanation empirically, such as how multi systems work together to determine and store the loss aversion parameter. Future research should compare the neurological data of the first several trials and the last several trials to test this hypothesis. Another limitation of our study involves the lack of simultaneous measurements of specific emotional processes. We only used a proven effective way to reduce the emotional response. The third limitation is that all the participants in our study are university students. Further studies should thus assess the extent to which our findings can be generalized to different subject samples. Perhaps more experienced decision makers may show a more consistent choice in these two treatments.

In summary, our findings indicate that when individuals make decisions for their future selves, loss aversion is reduced compared to when they make decisions for their current selves. Our findings endorsed the external validity of previous studies on the impact of emotions on loss aversion in a real world decision-making environment. This result emphasized the role of emotion on the reduced part of loss aversion, favoring multi-system theory. We also provide a possible explanation to reconcile the multi-system and single-system. Understanding this effect could enable us to make more specific and accurate predictions of economic behavior. Ultimately, making ourselves aware of this effect might help us overcome potentially disadvantageous decision biases.

## Author Contributions

All authors listed have made a substantial, direct and intellectual contribution to the work, and approved it for publication.

## Conflict of Interest Statement

The authors declare that the research was conducted in the absence of any commercial or financial relationships that could be construed as a potential conflict of interest.

## Footnotes

1 In fact, we considered whether we could use the within-subjects design, but we ultimately chose the between-subjects design. Precise control of the reference point is a critical part of our experiment, which cannot be easily implemented in a within-subjects design experiment. In a within-subjects design, the payoff of the previous task will affect the reference point in the subsequent task, which cannot be controlled as precisely as we expected. At the same time, because the decision task is relatively simple and we have the same tasks in different treatments, the carryover effect may easily affect the result (Harrison et al., 2005).

2 We chose class experiments rather than lab experiments for two reasons. The first reason is that the reference point is affected by many factors, such as rational expectations based on the opportunity cost of going to the lab. Even though some researchers argue that the money provided by the experimenters will be regarded as windfall money by the participants, if the participants intend to come to the lab to earn money and have a goal in mind, the rational expectation would be a reference point (Kõszegi and Rabin, 2006), especially when going to the lab requires opportunity costs and real effort. In a class, the money provided by experimenter will be regarded as a windfall gain more completely. The second reason is that resolution time manipulation is easier in the class experiment. We have two treatments, in which the time of resolution and payment will be made a month after the participants finish the choice tasks. In these two treatments, we should make sure that the participants believe that the future outcomes will be realized accordingly. A class experiment will be a guarantee for building such a belief.

3 Using a hypothetical payoff may give rise to doubts of whether the hypothetical condition is not appropriate to measure true risk preferences. However, our goal in measuring risk attitudes in this hypothetical section is not to calibrate the mean level of the subjects’ risk preferences but to control for individual variation. In the hypothetical section, the tasks finished by participants in all four treatments are exactly the same—the same choice problems and the same hypothetical payment. Therefore, it is reasonable to believe that this measure is useful for comparing the risk attitudes of the participants in the four treatments, although there is a hypothetical bias. This issue is discussed in detail in Brocklebank’s doctoral thesis (Brocklebank, 2012).
